# Native T1 high region and left ventricular ejection fraction recovery in patients with dilated cardiomyopathy

**DOI:** 10.1007/s10554-023-02888-w

**Published:** 2023-06-08

**Authors:** Mayu Yazaki, Takeru Nabeta, Yu Takigami, Yuko Eda, Kenji Maemura, Takumi Oki, Teppei Fujita, Yuichiro Iida, Yuki Ikeda, Shunsuke Ishii, Yusuke Inoue, Junya Ako

**Affiliations:** 1 Department of Cardiovascular Medicine, Kitasato University School of Medicine, 1-15-1 Kitasato, Minami-ku, Sagamihara, Kanagawa 252-0329 Japan; 2 Department of Cardiology, Leiden University Medical Center, Leiden, The Netherlands; 3 Department of Diagnostic Radiology, Kitasato University School of Medicine, Sagamihara, Kanagawa Japan

**Keywords:** Dilated cardiomyopathy, Cardiac magnetic resonance, T1 mapping, Left ventricular ejection fraction recovery

## Abstract

Native T1 mapping is used to assess myocardial tissue characteristics without gadolinium contrast agents. The focal T1 high-intensity region can indicate myocardial alterations. This study aimed to identify the association between the native T1 mapping including the native T1 high region and left ventricular ejection fraction (LVEF) recovery in patients with dilated cardiomyopathy (DCM). Patients with newly diagnosed DCM (LVEF of < 45%) who underwent cardiac magnetic resonance imaging with native T1 mapping were included in the analysis. Native T1 high region was defined as a signal intensity of > 5 SD in the remote myocardium. Recovered EF was defined as a follow-up LVEF of ≥ 45% and an LVEF increase of ≥ 10% after 2 years from baseline. Seventy-one patients met the inclusion criteria for this study. Forty-four patients (61.9%) achieved recovered EF. Logistic regression analysis showed that the native T1 value (OR: 0.98; 95% CI: 0.96–0.99; P = 0.014) and the native T1 high region (OR: 0.17; 95% CI: 0.05–0.55; P = 0.002), but not late gadolinium enhancement, were independent predictors of recovered EF. Compared with native T1 value alone, combined native T1 high region and native T1 value improved the area under the curve from 0.703 to 0.788 for predicting recovered EF. Myocardial damage, which was quantified using native T1 mapping and the native T1 high region were independently associated with recovered EF in patients with newly diagnosed DCM.

## Introduction

Dilated cardiomyopathy (DCM) is a myocardial disease commonly diagnosed based on impaired left ventricular ejection fraction (LVEF) and LV dilation [1]. LVEF recovery can be achieved with guideline-directed medical therapy (GDMT). Further, it is associated with favourable outcomes in patients with DCM [2, 3]. However, despite progression in GDMT, a significant number of patients with DCM do not achieve LVEF recovery [4].

Cardiac magnetic resonance (CMR) imaging is suitable for investigating cardiac properties, functions and geometry. Late gadolinium enhancement (LGE)-CMR imaging is a standard technique for evaluating myocardial fibrosis [5]. Moreover, myocardial fibrosis evaluated via LGE-CMR imaging has a significant prognostic value for predicting the incidence of cardiac events and LVEF recovery in patients with DCM [6]. However, the capability of LGE-CMR imaging in evaluating diffuse myocardial fibrosis is limited [7]. In addition, LGE is not applicable to patients with renal dysfunction because of the need for gadolinium-based contrast agents [8]. Biological tissues have a fixed T1 value. The T1 map, which reflects tissue characteristics, can be produced by measuring the T1 value of cardiac tissues pixel-by-pixel. However, LGE can only be utilised to qualitatively assess myocardial fibrosis. The novel T1 mapping technique can be used to quantitatively assess native T1 value and extracellular volume (ECV) fraction [9].

ECV is a surrogate measure of extracellular space and is equivalent to the myocardial volume of gadolinium contrast medium distribution. It is used to assess extracellular space, which is occupied by the extracellular matrix. Therefore, it can reflect diffuse myocardial fibrosis in the absence of protein deposition or oedema [7]. The native T1 value is a pre-contrast T1 value of the myocardium, which reflects its intracellular and extracellular interstitial components [10]. Native T1 mapping can be applied to evaluate myocardial properties without gadolinium contrast agents [11, 12]. A high native T1 value indicates cardiomyocyte oedema or a high interstitial component, which indicates myocardial alterations including fibrosis [9]. A previous study showed that a high native T1 value was a predictor of hospitalisation due to worsening heart failure (HF) and all-cause mortality in patients with DCM [13].  However, the association between native T1 mapping and LVEF recovery in patients with DCM is unclear. Recently, the heterogeneity of native T1 mapping of the myocardium has attracted attention because it can have an additive prognostic value for predicting cardiac events and LV reverse remodelling [14, 15]. Based on these results, not only global native T1 value but also regional differences in native T1 value can be clinically significant. A high native T1 value is associated with myocardial alterations such as fibrosis and inflammation. We hypothesised that the focal high-intensity region of native T1 mapping can reflect regional myocardial alterations. Thus, the current study aimed to investigate the association between native T1 mapping, including the regional intensity area and LVEF recovery in patients with DCM.

## Methods

### Participants

This single-centre, retrospective, observational study assessed patients with newly diagnosed DCM who underwent CMR imaging at Kitasato University Hospital in Japan between October 2016 and December 2019. DCM was defined as an LVEF of < 45% at baseline echocardiography. Patients with significant coronary artery disease (> 75% luminal stenosis on coronary angiography), cardiac amyloidosis, sarcoidosis, acute myocarditis, metabolic disorders, endocrine dysfunction, neuromuscular diseases, peripartum cardiomyopathy, organic heart valve disease, use of cardiotoxic drugs and alcohol abuse were excluded. The Ethics Committee of Kitasato University Medical approved this study and the use of clinically acquired data. Moreover, the need for a written informed consent was waived.

### CMR imaging acquisition

All CMR imaging procedures were performed with a 3.0T scanner (MAGNETOM Skyra, Siemens Healthineers, Erlangen, Germany) using a standard protocol. All images were obtained during breath holding at expiration. The cine images included the acquisition of three long-axis slices (two- and four-chamber) and a stack of short-axis slices covering the whole LV using a balanced steady-state free precession (bSSFP) sequence. Native T1 mapping was performed on a standard LV short-axis slice at the mid-ventricular level using the modified Look-Locker inversion recovery sequence combined with motion correction before contrast injection. The sequence parameters were as follows:

- For an RR interval of > 700 ms, repetition time (TR)/echo time (TE): 280.6/1.1 ms; matrix: 256 × 169; field of view (FOV): 360 × 307 mm^2^; flip angle (FA): 35°; bandwidth: 1085 Hz/pixel and slice thickness: 8 mm.
- For an RR interval of < 700 ms, TR/TE: 263.9/1.0 ms; matrix: 192 × 154; FOV: 360 × 307 mm^2^: FA: 35°; bandwidth: 1085 Hz/pixel and slice thickness: 8 mm.

LGE images were acquired 10–15 min after the intravenous injection of 0.2 mmol/kg gadolinium using the phase-sensitive inversion recovery bSSFP sequence. The sequence parameters were as follows: TR/TE, 2.7/1.1 ms; matrix, 224 × 180; FOV, 340 × 340 mm^2^; FA, 55°; bandwidth, 1,175 Hz/pixel; slice thickness, 8 mm and slice gap, 2 mm. Parallel imaging with GeneRalized Autocalibrating Partially Parallel Acquisitions was used for the sequence. The optimal inversion time (TI) was determined with a TI-scout sequence. This pulse sequence was used to determine optimal TI to null the signal intensity of the normal myocardium.

### CMR image analysis

**Fig. 1:**
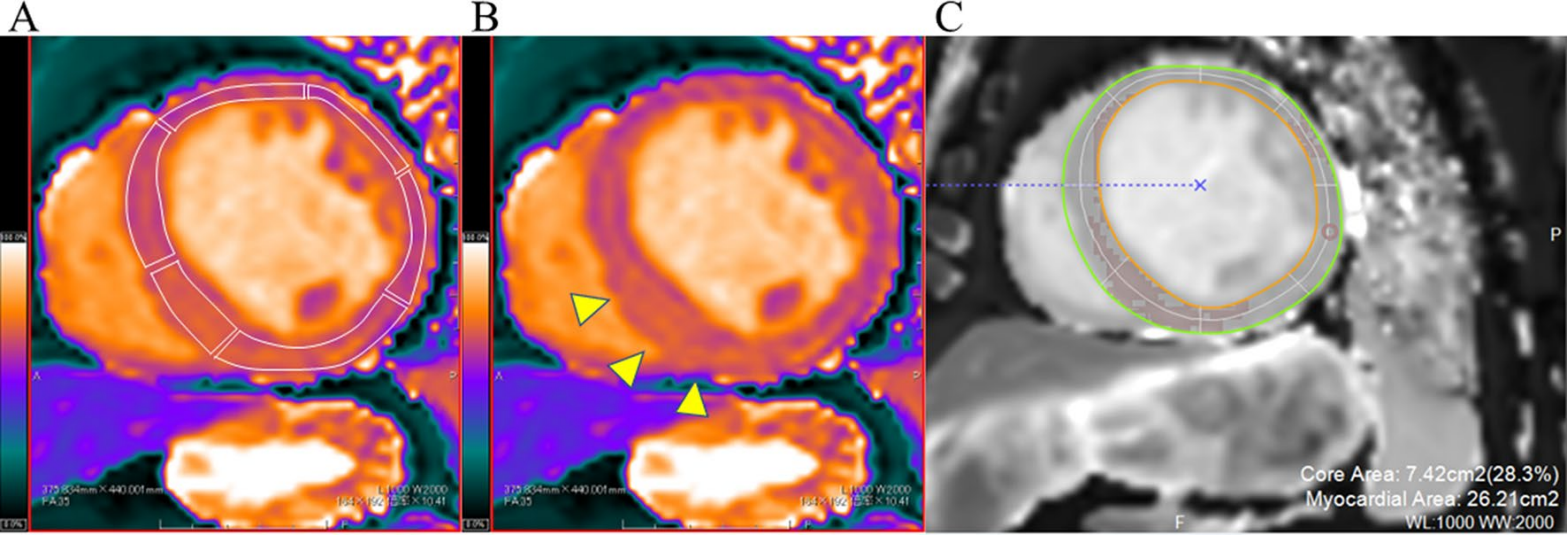
Evaluation of native T1 values and native T1 high region. **A** Native T1 values were determined by drawing the region-of-interest in each segment of the six regions in the mid-left ventricle. **B** The native T1 high region (arrowheads). **C** A region-of-interest of 0.5−1 cm^2^ was manually drawn in the visually normal myocardium. The native T1 high region ratio was defined as a signal intensity of > 5 SD in the remote myocardium and was expressed as a percentage of the left ventricular area

LV volumetric analysis was performed with a commercially available software (cvi42, version 4.1.8, Circle Cardiovascular Imaging, Calgary, Canada). The presence of LGE was defined by an experienced observer. The native T1 values were determined by drawing the region-of-interest (ROI) manually in each segment of the participants on a dedicated workstation with an ROI measuring tool (EV Insite, PSP Corporation, Tokyo, Japan), according to the six regions of the mid-ventricle in the 17-segment model [16]. The ROIs of all participants were drawn in a mid-wall region of the myocardium to minimise partial volume effects at the epicardial and endocardial borders (Fig. 1A) [17]. Similar to the measurement of the LGE area [18], the native T1 high region (Fig. 1B) was defined as a signal intensity of > 5 SD of the remote myocardium and was expressed as a percentage of the LV myocardium (native T1 high region ratio) using a commercially available software (Ziostation2, Ziosoft, Tokyo, Japan) (Fig. 1C).

### Clinical measurement and observation

At baseline, blood pressure, heart rate and laboratory parameters were evaluated upon undergoing CMR imaging and echocardiography was performed. Patients were treated according to the current guidelines.[19] Recovered EF was defined as an LVEF of ≥ 45% and an LVEF increase of ≥ 10% on follow-up echocardiography after 2 years from baseline.[4, 20]. The endpoint was hospitalisation due to worsening HF. The endpoint data were obtained from the patient’s medical records.

### Statistical analysis

Data were presented as mean ± standard deviation or as frequency (percentage). The Student’s *t*-test was used to compare continuous variables and the Mann–Whitney U test was utilised to evaluate non-normally distributed continuous variables. The Pearson’s chi-square test was applied to assessed categorical variables. A two-tailed P value of < 0.05 was considered statistically significant. Multivariate logistic regression analysis was performed to identify variables correlated with recovered EF among all baseline variables. The univariate analysis included potential covariates affecting LVEF recovering, as shown in a previous study [21]. Clinical variables with a P value of < 0.2 in the univariate analysis were examined in the multivariate analysis. The event-free survival curves were drawn using the Kaplan–Meier method and were compared using the log-rank test. Receiver-operating characteristic (ROC) curve analysis was performed to compare the discriminative power of predicting recovered EF between the native T1 value and the native T1 high region. Statistical analyses were performed using JMP version 14 (SAS Institute, Cary, NC, the USA) and R (version 4.1.1, R Project for Statistical Computing).

## Results

### Patient selection and baseline characteristics

**Fig. 2:**
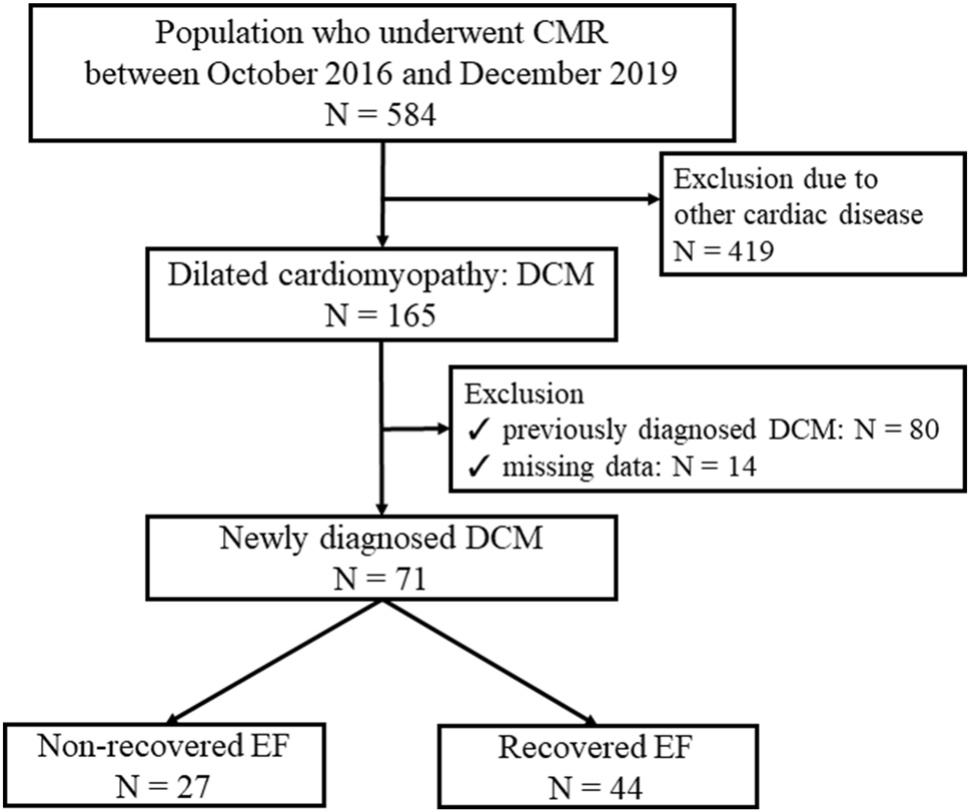
Patient selection. * CMR* cardiac magnetic resonance, *EF* ejection fraction

In total, 71 patients met the inclusion criteria of this study. After 2 years from baseline, 44 patients (61.9%) achieved recovered EF (Fig. 2). Table 1 shows the baseline characteristics of the patients. Age, sex, blood pressure, comorbidity, medications, electrocardiogram, laboratory values and echocardiography results did not significantly differ between the non-recovered EF group and the recovered EF group. Table 2 depicts the baseline CMR imaging data. The LV volumes and the presence of LGE on CMR imaging did not significantly differ between the two groups. The non-recovered EF group had a significantly higher native T1 value than the recovered EF group (1329.5 ± 49.8 ms vs. 1296.3 ± 37.1 ms, P = 0.002). The non-recovered EF group was more likely to present with a native T1 high region than the recovered EF group (77.8% vs. 38.4%, P = 0.001). Furthermore, the non-recovered EF group had a significantly higher native T1 high region ratio than the recovered EF group (23.5 ± 16.5% vs. 8.8 ± 12.0%, P < 0.001).

**Table 1:** Characteristics of the patients at baseline

Variables	Non-recovered EF group (n = 27)	Recovered EF group (n = 44)	P value
Age (years)	59.0 ± 12.7	57.7 ± 12.2	0.679
Male, n (%)	21 (77.8)	30 (68.2)	0.383
BSA (m^2^)	1.69 ± 0.21	1.70 ± 0.24	0.804
Systolic blood pressure (mmHg)	115.8 ± 14.6	120.1 ± 17.8	0.299
Diastolic blood pressure (mmHg)	72.3 ± 13.8	74.4 ± 15.4	0.550
Heart rate (beats/min)	70.6 ± 9.7	74.8 ± 16.4	0.223
Comorbidity, n (%)			
Diabetes mellitus	5 (18.5)	7 (15.9)	0.776
Hypertension	11 (40.7)	21 (47.7)	0.566
Medications, n (%)			
Beta-blocker	27 (100.0)	43 (97.7)	0.430
ACEi/ARB	27 (100.0)	44 (100.0)	-
MRA	11 (40.7)	26 (59.1)	0.133
Furosemide	13 (48.2)	28 (63.6)	0.200
Electrocardiogram, n (%)			
Atrial fibrillation	4 (14.8)	7 (15.9)	0.902
CLBBB	7 (25.9)	5 (11.4)	0.112
Laboratory values			
Haemoglobin level (mg/dL)	14.4 ± 1.3	14.4 ± 1.5	0.900
Haematocrit (%)	43.4 ± 3.8	43.0 ± 4.4	0.679
Total bilirubin level (mg/dL)	0.66 ± 0.23	0.73 ± 0.32	0.388
Serum creatinine level (mg/dL)	0.95 ± 0.21	0.95 ± 0.17	0.871
eGFR (mL/min/1.73 m^2^)	63.1 ± 16.5	60.8 ± 12.4	0.513
BNP level (pg/mL)	365.7 ± 410.1	307.6 ± 310.5	0.790
Troponin T level (ng/mL)	0.03 ± 0.02	0.03 ± 0.03	0.509
Echocardiography			
LAD (mm)	41.3 ± 6.7	41.2 ± 7.9	0.962
LVDd (mm)	64.0 ± 8.5	61.8 ± 9.1	0.313
LVDs (mm)	51.5 ± 8.6	53.3 ± 9.3	0.316
LVEF (%)	31.2 ± 9.1	30.2 ± 7.8	0.616
E/e’	13.9 ± 7.1	11.7 ± 4.4	0.145
TRPG (mmHg)	25.3 ± 12.8	23.2 ± 8.7	0.459
MR grade ≥ 3, n (%)	6 (22.2)	9 (20.5)	0.859

*ACEi/ARB* angiotensin-converting enzyme inhibitor/angiotensin-receptor blocker, *BNP* brain natriuretic peptide, *BSA* body surface area, *CLBBB* complete left bundle branch block, *eGFR* estimated glomerular filtration rate, *LAD* left atrial dimension, *LVDd* left ventricular end-diastolic dimension, LVDs left ventricular end-systolic dimension, *LVEF* left ventricular ejection fraction, *MR* mitral regurgitation, *MRA* mineralocorticoid receptor antagonist, *TRPG* transtricuspid pressure gradient

Categorical variables were presented as numbers (percentages) and continuous variables are shown as mean ± standard deviation

**Table 2:** Cardiac magnetic resonance at baseline

	Non-recovered EF (N = 27)	Recovered EF (N = 44)	P value
LVEDV (mL)	289.2 ± 77.3	264.2 ± 89.8	0.234
LVEDVI (mL/m^2^)	170.0 ± 45.8	152.9 ± 42.0	0.111
LVESV (mL)	213.1 ± 81.4	196.8 ± 84.7	0.429
LVESVI (mL/m^2^)	128.5 ± 47.7	115.4 ± 42.1	0.231
LVEF (%)	25.9 ± 8.7	27.0 ± 9.0	0.606
Cardiac output (L/min)	5.0 ± 1.5	4.8 ± 1.3	0.615
Cardiac index (L/min/m^2^)	2.9 ± 0.7	2.8 ± 0.6	0.550
Presence of LGE, n (%)	16 (59.3)	18 (40.9)	0.133
Anterior native T1 value (ms)	1297.2 ± 47.4	1281.3 ± 43.9	0.155
Anteroseptal native T1 value (ms)	1336.2 ± 41.0	1316.5 ± 36.5	0.038
Inferoseptal native T1 value (ms)	1343.7 ± 52.4	1315.8 ± 39.2	0.013
Inferior native T1 value (ms)	1353.9 ± 105.2	1304.5 ± 69.1	0.019
Inferolateral native T1 value (ms)	1341.7 ± 116.1	1278.9 ± 50.1	0.002
Anterolateral native T1 value (ms)	1304.3 ± 48.2	1280.8 ± 48.9	0.053
Average native T1 value (ms)	1329.5 ± 49.8	1296.3 ± 37.1	0.002
Presence of native T1 high region, n (%)	21 (77.8)	17 (38.4)	0.001
Native T1 high region ratio (%)	23.5 ± 16.5	8.8 ± 12.0	< 0.001

*LGE* late gadolinium enhancement, *LVEDV* left ventricular end-diastolic volume, *LVEDVI* left ventricular end-diastolic volume index, *LVEF* left ventricular ejection fraction, *LVESV* left ventricular end-systolic volume, *LVESVI* left ventricular end-systolic volume index

Categorical variables are shown as numbers (percentages) and continuous variables are shown as mean ± standard deviation

**Fig. 3:**
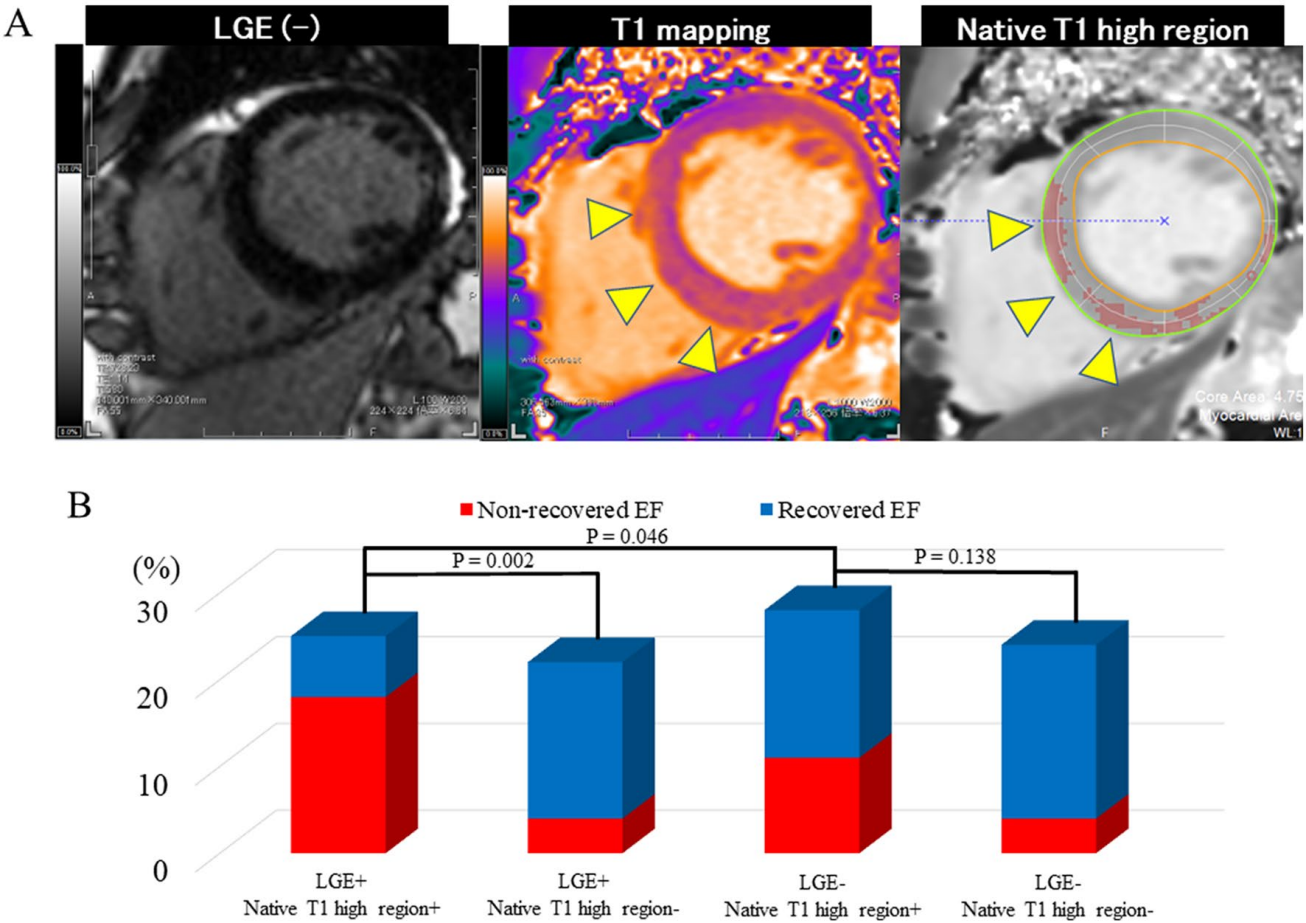
Native T1 high region and LGE. ** A** Representative case. The patient presented with the native T1 high regions (arrowheads) without LGE. **B** Native T1 high region and LGE proportions. *EF* ejection fraction, *LGE* late gadolinium enhancement

In total, 20 (28.2%) patients presented with native T1 high regions despite the absence of LGE (Fig. 3A). In patients with LGE, the non-recovered EF group with the native T1 high region was significantly more frequently observed than those without (P = 0.002). In patients without LGE, the non-recovered EF group with the native T1 high region also tended to be more frequently observed than those without (P = 0.138) (Fig. 3B).

### CMR imaging findings associated with recovered EF and prognosis

**Fig. 4:**
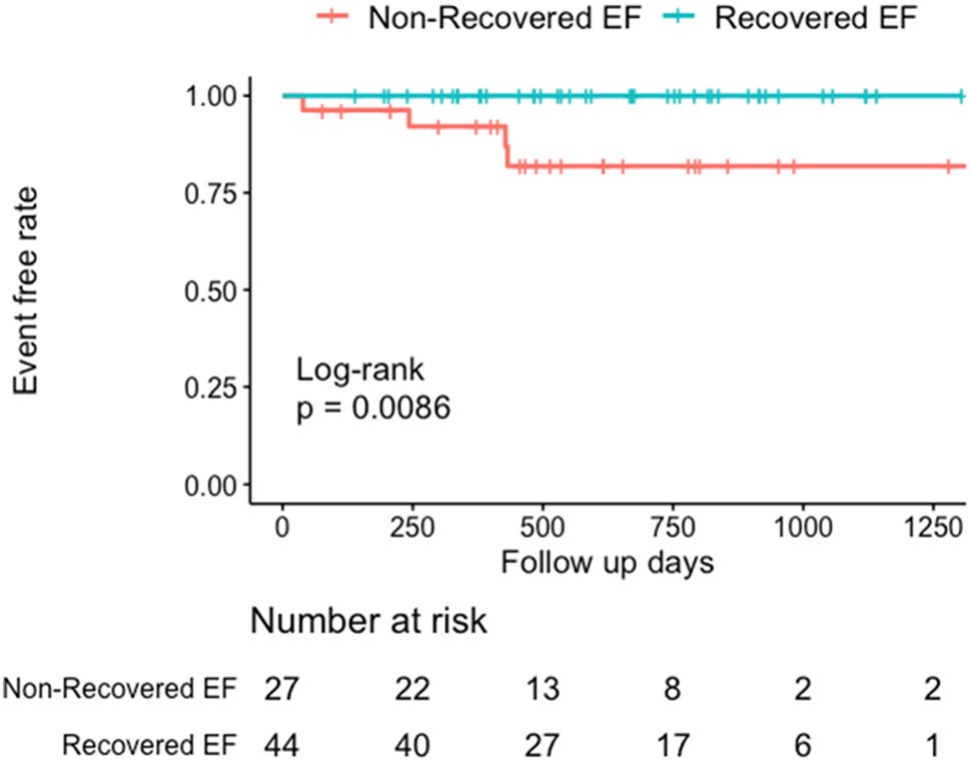
Kaplan–Meier curves of hospitalisation due to heart failure. Based on the Kaplan**–**Meier curves, patients with the non-recovered EF group had a significantly higher risk of hospitalisation due to heart failure than those with the recovered EF group. *EF* ejection fraction

According to the results of the univariate analysis, mineralocorticoid receptor antagonist, complete left bundle branch block, LGE, native T1 value and native T1 high region were included in the multivariate model. Two multivariate models, one containing the native T1 value and one containing the native T1 high region, were constructed. In model 1, the native T1 value (odds ratio [OR]: 0.98; 95% confidential interval [CI]: 0.96–0.99; P = 0.014) was considered an independent factor associated with recovered EF. In model 2, the presence of the native T1 high region (OR: 0.17; 95% CI: 0.05–0.55; P = 0.002) was identified as an independent factor associated with recovered EF (Table 3). Based on the Kaplan–Meier curve, the non-recovered EF group had a significantly higher risk of hospitalisation due to HF than the recovered EF group (Fig. 4). Seven of the complete left bundle branch block patients underwent cardiac resynchronization therapy insertion, with no significant difference between the two groups [the non-recovered EF group: 3 (11.1%) vs. the recovered EF group: 4 (9.1%); P = 0.782].

**Table 3:** Univariate and multivariate analyses of LVEF recovery

	Univariate analysis			Multivariate analysis (model 1)			Multivariate analysis (model 2)		
	OR	95% CI	P value	OR	95% CI	P value	OR	95% CI	P value
Age	0.99	0.95–1.03	0.674						
Male sex	0.61	0.20–1.85	0.385						
BSA	1.32	0.15–11.4	0.801						
Systolic blood pressure	1.01	0.98–1.04	0.296						
Heart rate	1.02	0.99–1.06	0.224						
Beta-blocker	< 0.99		0.999						
ACEi/ARB	< 0.99		0.999						
MRA	2.10	0.79–5.57	0.136	3.02	0.99–9.24	0.052	1.97	0.64–6.07	0.237
CLBBB	0.37	0.10–1.30	0.121	0.60	0.14–2.58	0.496	0.30	0.07–1.35	0.107
Haemoglobin level	0.98	0.70–1.37	0.898						
Serum creatinine level	0.80	0.06–11.4	0.869						
BNP level	0.99	0.98–1.00	0.498						
LVEF	0.98	0.93–1.04	0.610						
LAD	0.99	0.93–1.06	0.961						
MR grade ≥ 3	0.90	0.28–2.89	0.859						
LVEDV	0.99	0.98–1.00	0.239						
LVESV	0.99	0.98–1.00	0.428						
LGE	0.48	0.18–1.26	0.136	0.54	0.18–1.61	0.269	0.42	0.13–1.29	0.121
Native T1 value	0.18	0.06–0.54	0.001	0.98	0.96–0.99	0.014			
Native T1 high region	0.98	0.97–0.99	0.007				0.17	0.05–0.55	0.002

*ACEi/ARB* angiotensin-converting enzyme inhibitor/angiotensin-receptor blocker, *BNP* brain natriuretic peptide, *BSA* body surface area, *CLBBB* complete left bundle branch block, *LAD* left atrial dimension, *LGE* late gadolinium enhancement, *LVEDV* left ventricular end-diastolic volume, *LVEF* left ventricular ejection fraction, *LVESV* left ventricular end-systolic volume, *MR* mitral regurgitation, *MRA* mineralocorticoid receptor antagonist

**Fig. 5:**
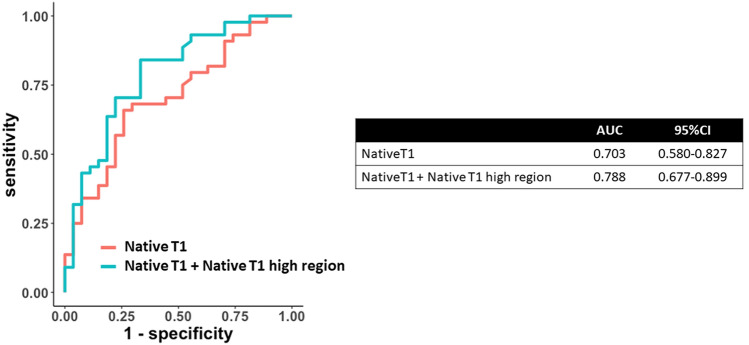
Receiver-operating characteristic curve for predicting LVEF recovery. The receiver-operating characteristic curves were used to compare the discriminative power of predicting recovered left ventricular ejection fraction between the native T1 value and the native T1 high region

Figure 5 depicts the ROC curves of the native T1 value and the native T1 high region for predicting recovered EF. Compared with native T1 value alone, combined native T1 high region and native T1 value improved the area under the curve from 0.703 to 0.788 for predicting recovered EF.

## Discussion

### Major findings

The current study showed the clinical significance of native T1 mapping in patients with newly diagnosed DCM. There were no significant differences in LV volumes and the presence of LGE between patients with recovered EF and those without. However, patients without recovered EF had a higher native T1 value than those with recovered EF. Furthermore, both the native T1 value and the presence of the native T1 high region were independently associated with recovered EF. The native T1 high region had an incremental prognostic value for predicting recovered EF. Thus, the native T1 high region and the native T1 value might be important markers for predicting recovered EF in patients with DCM.

### High native T1 value and LVEF recovery

A high native T1 value indicates oedema (increased tissue water content caused by different factors including inflammation) and high interstitial components (e.g. fibrosis and amyloid deposition) [9]. In the current study, the non-recovered EF group had a significantly higher native T1 value than the recovered EF group. Hence, fibrosis progression was correlated with the presence or absence of LVEF improvement. These results are consistent with those of two previous studies, which reported that patients without LVEF recovery had a significantly higher native T1 value and ECV than those with LVEF recovery.[14, 22] In contrast, another study found that ECV and T2 values but not native T1 values were associated with LV reverse remodelling [23]. This discrepancy might be explained by differences in the definition of LVEF recovery and the study cohort. For example, this study only included 58% of patients with newly diagnosed DCM. However, in the current study, all patients were newly diagnosed with DCM. This difference might affect differences in the prognostic factors of LVEF recovery because the HF duration is a key factor of LVEF recovery [24].

Both native T1 and ECV fractions are correlated with histological collagen volume fractions. ECV quantifies the interstitial uptake of gadolinium contrast agent relative to the plasma. Native T1 mapping can be an effective alternative option to ECV measurement if a shorter scan time and non-contrast material requirements are considered [12]. Thus, native T1 mapping can be a more convenient method for identifying patients with or without LVEF recovery after GDMT.

### Native T1 high region and LGE

LGE is the gold standard method for assessing focal myocardial damage representative of fibrosis [25]. LGE is associated with poor LVEF recovery in patients with DCM [6]. Conversely, similar to the current research, some studies showed that LGE was not a predictor of LVEF recovery [22, 26]. The reason for this discrepancy is unclear. However, the differences in cohorts and the number of events might be a cause.

In the current study, the native T1 high region was associated with LVEF recovery. Two previous studies revealed that the native T1 value of the segments with LGE was higher than that of the segments without LGE in patients with DCM [27, 28]. However, the association between T1 values per segment and the fraction of segments with LGE were modest [27]. In our research, the native T1 high region was observed in the segments without LGE. Therefore, the native T1 high region reflects focal myocardial alterations. However, it is not necessarily consistent with LGE. This discrepancy might be attributed to the fact that LGE may primarily indicate myocardial fibrosis and a high native T1 represents oedema and high interstitial components [9]. Moreover, Dass et al. showed that T1 mapping and LGE measurement were partly overlapping. Nevertheless, there were distinct myocardial pathologies [27]. Furthermore, T1 mapping facilitates direct myocardial signal quantification on a standardised scale. The advantages of T1 mapping can allow a better characterisation of myocardial tissue compared with LGE [25]. Therefore, the native T1 high region is more effective in identifying focal myocardial damage than LGE.

In a previous study, the native T1 value significantly decreased after GDMT. This finding indicates reduced collagen volume fraction and serum myocardial collagen accumulation index in patients with DCM after GDMT [23]. The current research did not assess changes in the native T1 value and the native T1 high region over time. Hence, further studies on this topic should be performed.

### Clinical implications

Native T1 mapping can be performed on all patients eligible for CMR imaging because it does not require gadolinium contrast agents. In addition, the native T1 high region had an additive predictive value for predicting LVEF recovery. In relation to this reason, it might be helpful to focus on the native T1 value and the native T1 high region in determining appropriate treatment strategies in patients with newly diagnosed DCM.

### Study limitations

The current study had several limitations that must be acknowledged. First, this was a single-centre study with a limited number of patients. This could have resulted in patient selection bias and a lower statistical power. Second, only patients undergoing CMR imaging were included in the analysis, which could have posed a risk of bias. Third, the statistical power was limited because of the small number of events. Fourth, T1 mapping images of LV were acquired only at the mid-ventricular level. Therefore, the whole LV myocardium could not be evaluated.

## Conclusion

The quantitative value of native T1 mapping and the presence of a high-signal intensity region were independent predictors of LVEF improvement in patients with newly diagnosed DCM. Combined native T1 value and native T1 high region was more accurate in predicting LVEF improvement than native T1 value alone. Hence, T1 mapping might be useful in identifying therapeutic options for patients with DCM.

## Data Availability

The datasets used and analysed during the current study are available from the corresponding author on reasonable request.
